# 

**Published:** 1916-12-30

**Authors:** 


### CONTENTS.

PAGE

**A Memorable Christmas Day** ... ... ... 255

**Practitioners and Earning Power** ... ... 255

**Hospital and Institutional News** ... ... ... 257

Work for Convalescent Soldiers—Death of the Founder of the Hospital for Hip Disease, Sevenoaks—A New Canadian Convalescent War Hospital—More Canadian Hospital Developments—Medical Men as Ministers—German Submarine Benefits Hospital—A Physician's Legacy to His Hospital—A Record of Activity—The Expansion of Hospitals: The New Phase—The Institutional Meatless Day—The Premier and Welsh Ambulance Work—Architecture and Public Health—The New Hawkmoor Sanatorium—"Tubs for Tommies"—The Problem of Premises—The Cavell Statue—Vegetable Cultivation for Hospitals—Hospital Servant Problem—"The Fifth"—This Week's Drug Market—To Our Readers.

**The Peter Bent Brigham Hospital, Boston** ... 261

**The School Doctor** ... ... ... ... ... 263

The School Doctor and the Compulsion of Parents.

**Macedonia in War-Time** ... ... ... ... 264

By an Officer of the R.A.M.C. and the Author of "Nature Notes."

**The Metropolitan Hospital Sunday Fund** ... 265

Annual Meeting of Constituents.

**The Responsibilities of Voluntary Hospitals**... 266

**Hospital Architecture and Construction** ... 270

Royal Albert Edward Infirmary, Wigan.

PAGE

**Christmas in the Hospitals**... ... ... ... ... 267

St. Thomas's Hospital—St. George's Hospital—St. Mary's Hospital—Hampstead Military Hospital—South London Hospital for Women: Its First Christmas—The Leicester Hospitals.

**The Matrons' and Sisters' Department** ... 271

The Editor and His Readers.

Round the Hospitals.

Queen Alexandra in her Hospital—Nursing Opinion and the College—Speed up Registration—Miss Darbyshire's New Appointment—The Spirit of the Nation—Important to Poor-Law Nurses.

**The Editor's Letter-Box** ... ... ... ... 273

Is there a Shortage in the Supply of Nurses?—Accident to Mr. A. W. Faire, Deputy-Lieutenant of Leicestershire—How Increases in Railway Fares affect the Devonshire Hospital.

**Gifts to Hospitals** ... ... ... .. ... 273

**Patriotism of the Nurse-Training Schools** ... 274

XI. War Work in Glasgow Institutions.

**Matrons' and Sisters' Appointments** ... ... iv

**Medical Appointments** ... ... ... ... iv

**Parliament and the Profession** ... ... ... iv

**The Institutional Worker** ... ... (Suppl.) 1

Guy's Hospital Pension Scheme.

The Feeding of Wounded Soldiers.

Limiting Flag Days.

Question for December.

Questions Invited.

